# Effect of the Blooming Artifact Reduction filter on the detection of voids in root canal fillings using cone-beam computed tomography images

**DOI:** 10.1590/0103-644020256780

**Published:** 2026-01-30

**Authors:** Airton Oliveira Santos-Junior, Rocharles Cavalcante Fontenele, Jáder Camilo Pinto, André Ferreira Leite, Fernanda Ferrari Esteves Torres, Karina Ines Medina Carita Tavares, Marcelo Gonçalves, Juliane Maria Guerreiro-Tanomaru, Mário Tanomaru-Filho

**Affiliations:** 1 Department of Restorative Dentistry, São Paulo State University (UNESP), School of Dentistry, Araraquara, São Paulo, Brazil; 2 OMFS IMPATH Research Group, Department of Imaging and Pathology, Faculty of Medicine, University of Leuven, Leuven, Belgium; 3 Department of Stomatology, Public Health and Forensic Dentistry, Division of Oral Radiology, School of Dentistry of Ribeirão Preto, University of São Paulo (USP), Ribeirão Preto, São Paulo, Brazil; 4 Department of Dentistry, Centro Universitário Presidente Antônio Carlos (UNIPAC), Barbacena, Minas Gerais, Brazil; 5 Department of Dentistry - Centro Universitário Presidente Tancredo de Almeida Neves (UNIPTAN), São João del Rei, Minas Gerais, Brazil; 6 Department of Dentistry, University of Brasília (UnB), Campus Darcy Ribeiro, Asa Norte, Brasília, Distrito Federal, Brazil; 7 Department of Diagnosis and Surgery, São Paulo State University (UNESP), School of Dentistry, Araraquara, São Paulo, Brazil

**Keywords:** blooming artifact, cone-beam computed tomography, micro-computed tomography, root canal obturation

## Abstract

This study evaluated the effectiveness of the blooming artifact reduction (BAR) filter (e-Vol DX Software, CDT, Bauru, SP, Brazil) for detecting voids in root canals filled with various sealers, using two CBCT devices and micro-CT as a reference. Twenty mandibular incisors with long-oval root canals were filled with AH Plus Jet (AHPJ) or Bio-C Sealer (BCS). Each tooth was placed in the mandibular alveolar socket of a dry skull, covered with Mix-D material, and scanned with VeraView X800 and OP300 Maxio CBCT devices. Micro-CT images served as a reference. Three calibrated examiners assessed the CBCT scans, without and with the BAR filter (intensity level 1 for OP300 Maxio and level 3 for VeraView X800), for void detection using a 5-point scale. Diagnostic values (area under the receiver operating characteristic (ROC) curve, sensitivity, and specificity) were compared using multi-way analysis of variance (p=0.05). Intra- and inter-observer agreement was evaluated using the weighted Kappa index. For OP300, using BAR filter 1 significantly improved ROC curve values for detecting voids in the apical third with AHPJ and BCS (p<0.05). It also increased specificity for detecting voids in the apical third with AHPJ (p<0.05). For VeraView X800, the ROC curve and sensitivity values were unaffected (p>0.05), but using BAR filter 3 significantly enhanced specificity for detecting voids in the cervical/middle thirds with BCS (p<0.05). The diagnostic performance of the BAR filter is CBCT-device dependent. In OP300 scans, it enhances the detection of voids in the apical third with AHPJ and BCS. In VeraView X800 scans, it improves specificity for detecting voids in the cervical/middle thirds with BCS.

## Resumo

Este estudo avaliou a efetividade do filtro *Blooming Artifact Reduction* (BAR) (e-Vol DX Software, CDT, Bauru, SP, Brasil) para a detecção de espaços vazios em canais radiculares obturados com diferentes cimentos, utilizando dois aparelhos de TCFC e a micro-CT como referência. Vinte incisivos inferiores com canais radiculares ovais longos foram obturados com AH Plus Jet (AHPJ) ou Bio-C Sealer (BCS). Cada dente foi posicionado no alvéolo mandibular de um crânio seco, recoberto com material Mix-D e escaneado nos aparelhos de TCFC VeraView X800 e OP300 Maxio. As imagens de micro-CT foram utilizadas como referência. Três examinadores calibrados avaliaram as imagens de TCFC, sem e com aplicação do filtro BAR (intensidade nível 1 para o OP300 Maxio e nível 3 para o VeraView X800), para detecção de espaços vazios usando uma escala de cinco pontos. Os valores diagnósticos (área sob a curva ROC, sensibilidade e especificidade) foram comparados por meio de análise de variância multifatorial (p=0,05). A concordância intra e interobservador foi avaliada pelo índice Kappa ponderado. No OP300, o uso do filtro BAR 1 aumentou significativamente os valores da curva ROC para detecção de espaços vazios no terço apical com AHPJ e BCS (p<0,05), além de elevar a especificidade para detecção no terço apical com AHPJ (p<0,05). No VeraView X800, os valores da curva ROC e da sensibilidade não foram afetados (p>0,05), porém o uso do filtro BAR 3 aumentou significativamente a especificidade para detecção de espaços vazios nos terços cervical/médio com BCS (p<0,05). O desempenho diagnóstico do filtro BAR é dependente do aparelho de TCFC. Nos exames do OP300, ele melhora a detecção de espaços vazios no terço apical com AHPJ e BCS. Nos exames do VeraView X800, aumenta a especificidade para detecção nos terços cervical/médio com BCS.

**Figure:**
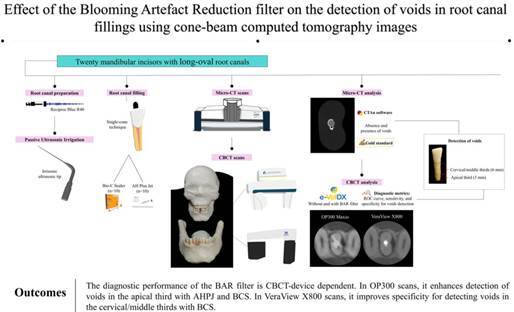

## Introduction

Three-dimensional obturation of the root canal system is essential for the success of endodontic treatment ^1^. However, root canal morphology significantly influences obturation quality ^2^ ^,^ ^3^. Filling long-oval canals is particularly challenging due to their buccolingual dimensions, increasing the likelihood of voids ^3^ ^,^ ^4^. Voids are spaces within the filling material that harbor microorganisms, allowing bacterial growth, reinfection, and treatment failure ^5^. Clinically, detecting voids in obturations is essential ^1^ ^,^ ^2^ ^,^ ^3^ ^,^ ^4^ ^,^ ^5^ ^,^ ^6^. In suspected treatment failure, evaluating obturation quality is key to planning retreatment or identifying failure causes, potentially avoiding unnecessary tooth extractions ^6^.

Cone-beam computed tomography (CBCT) has become the primary three-dimensional (3D) imaging modality for endodontic diagnosis and treatment planning, overcoming the inherent limitations of periapical radiography in detecting voids within root canal fillings ^7^ ^,^ ^8^ ^9^. However, CBCT scans can be compromised by high-density materials with elevated atomic numbers, such as endodontic sealers, gutta-percha, and intracanal metal posts, which produce artifacts that degrade image quality and affect diagnostic image evaluation ^10^ ^,^ ^11^ ^,^ ^12^.

Bio-C Sealer (Angelus, Londrina, PR, Brazil) is a bioceramic endodontic sealer containing zirconium oxide as a radiopacifier, with radiopacity values ranging from 5.5 to 7.12 millimeters of aluminum (mm Al) ^13^ ^,^ ^14^. In contrast, AH Plus Jet (Dentsply DeTrey, Konstanz, Germany) is an epoxy resin-based root canal sealer that combines zirconium with calcium tungstate, resulting in high radiopacity (exceeding 9 mm Al) ^13^ ^,^ ^14^. These differences in radiopacity values can lead to distinct artifacts on CBCT scans ^11^. One prominent artifact is volumetric distortion, commonly referred to as blooming, which causes the digitized volume to be overestimated, thereby compromising image quality and diagnostic accuracy ^10^ ^,^ ^11^ ^,^ ^12^.

Although it may not be feasible to eliminate artifacts completely, various strategies can optimize CBCT image quality. These include pre-processing techniques such as activating the metal artifact reduction (MAR) tool ^15^ or applying post-processing filters ^16^. Among these, the blooming artifact reduction (BAR) filter, integrated into the e-Vol DX software (CDT Software, Bauru, SP, Brazil), was specifically developed to reduce volumetric distortion artifacts ^17^. The BAR filter operates by enhancing brightness and contrast, leveraging the maximum dynamic range of Digital Imaging and Communication in Medicine (DICOM) files ^18^. This improvement makes the BAR filter a promising tool for better detection of voids within filling materials on CBCT scans ^19^. However, to the best of our knowledge, there is no evidence on how the BAR filter impacts the diagnostic accuracy in detecting voids in endodontically treated teeth.

Therefore, the aim of this study was to evaluate the impact of the BAR filter on detecting voids in long-oval root canals of mandibular incisors filled with AH Plus Jet or Bio-C Sealer on CBCT scans from different devices, using micro-computed tomography (micro-CT) images as reference. The null hypothesis was that the use of the BAR filter would not affect void detection, regardless of the CBCT device or type of sealer.

## Materials and methods

### Ethical implications and sample selection

After approval by the Institutional Ethics Committee (CAAE #41916720.7.0000.5416), human mandibular central incisors extracted for different purposes unrelated to the present investigation, such as periodontal disease, implant planning, or oral and maxillofacial surgical procedures, were collected from an institutional tooth bank. Two calibrated examiners (A.O.S.J. and K.I.M.C.T.) performed independent radiographic and micro-CT evaluations for specimen screening, and any discrepancies were resolved by consensus. Periapical radiographs were obtained using a digital RVG system (RVG 6100; Kodak Dental Systems, Rochester, NY) for initial screening to exclude teeth with previous endodontic treatment, root resorption, incomplete root formation, extensive coronal destruction, or open apices. Only teeth with a single root canal (Vertucci type I) ^20^ were included. Subsequently, the selected teeth were scanned using a micro-CT system with a voxel size of 24.5 µm (SkyScan 1272; Bruker, Kontich, Belgium) to confirm the internal anatomy and identify long-oval canals. Teeth were considered to have a long-oval cross-section when the buccolingual diameter was 2 to 4 times larger than the mesiodistal diameter ^21^ at 9 mm from the radiographic apex ^22^. Based on these evaluations, 20 human mandibular central incisors were selected for the study.

### Root canal preparation

Conventional access cavities were made with a #1012 diamond bur, followed by initial exploration of the root canals with a #10 K-file (Dentsply Sirona, Ballaigues, Switzerland). The working length (WL) was determined by subtracting 1.0 mm from the measurement obtained when the tip of the #10 K-file was visible at the apical foramen. Root canal preparation was performed with Reciproc Blue R40 instruments (VDW GmbH, Munich, Germany), driven by the X-Smart Plus electric motor (Dentsply Sirona), set to the “Reciproc All” function according to the manufacturer's instructions. The instrument was gradually introduced into each root canal, with in-and-out movements in the cervical, middle, and apical thirds, up to the WL. Two brushing movements were then made towards the buccal and lingual walls to reach the flattened areas of the root canal ^23^. For the irrigation step, 6 mL of 2.5% sodium hypochlorite (NaOCl; Ciclo Farma, Serrana, SP, Brazil) was used, 2 mL for each third (cervical, middle, and apical), using a 30-G NaviTip needle (Ultradent, South Jordan, UT) adapted to a 5 mL syringe (Ultradent), positioned 2 mm short of the WL.

To optimize cleaning after preparation with Reciproc Blue R40, all root canals underwent passive ultrasonic irrigation (PUI) protocol using the Irrisonic ultrasonic tip (Helse Ultrasonic, Santa Rosa de Viterbo, São Paulo, Brazil). According to the manufacturer's instructions, the Irrisonic tip was activated at 10% power and 50 Hz frequency with the Newtron® Booster ultrasonic device (ACTEON, North America, NJ, United States). The tip was positioned 2 mm short of the WL within each root canal and gently moved in-and-out without contacting the canal walls. Each root canal was subjected to three agitation cycles of 20 seconds each. During the first and third cycles, 2 mL of 2.5% NaOCl was used, while the second cycle involved 2 mL of 17% EDTA. Following these cycles, a final irrigation was performed with 5 mL of distilled water per sample, following the protocol outlined by Pinto et al. ^24^.

### Root canal filling

After root canal preparation, the specimens were divided into two experimental groups (n=10 each) using simple stratified randomization. This approach ensured that the post-preparation root canal volumes (mm³), measured using CTAn software (v.1.15.4.0, Bruker), were comparable across groups to maintain consistency during the obturation phase. 

For obturation, all root canals were filled using the single-cone technique with either AH Plus Jet or Bio-C Sealer (n=10 each). R40 gutta-percha principal cones (VDW GmbH) were selected using a profilometer device (Profile Projector Nikon model 6C-2; Nikon, Tokyo, Japan). Digital radiographs were taken in the mesiodistal and buccolingual directions to confirm the adaptation of the gutta-percha cones at WL. For the AH Plus Jet sealer, it was inserted into the root canals using a Lentulo spiral #40, coupled with a low-speed motor (Micromotor N270 and contra-angle, Dabi-Atlante, Ribeirão Preto, SP, Brazil), positioned 2 mm short of the WL. In addition, #40 K-files were also used with in-and-out movements up to the WL, in alignment with the methodology described by Tavares et al. ^3^.

In canals filled with Bio-C Sealer, the sealer was injected using syringes with plastic needles positioned 4 mm from the WL, following the manufacturer’s recommendations. The syringe plunger was gently pressed until the Bio-C Sealer flowed back to the pulp chamber, indicating complete filling of the root canals. R40 gutta-percha cones, coated with the respective sealers according to each experimental group, were then inserted into the canals up to the WL. Excess filling material was removed and vertically condensed with a 2 heat plugger (Golgran, Sao Caetano do Sul, SP, Brazil). Digital radiographs were taken in the mesiodistal and buccolingual directions to confirm adequate filling of all samples. 

Finally, the teeth were restored with Coltosol (Vigodent, Rio de Janeiro, Brazil) and stored in an oven at 37 °C and 95% humidity for 7 days to allow complete setting of the endodontic sealers.

### Micro-CT acquisition

To obtain a gold standard reference for comparisons with CBCT scans, each tooth was individually scanned using a micro-CT device (SkyScan 1272). The scanning parameters were set as follows: voxel size of 9 μm, a 1 mm aluminum filter, exposure time of 2500 ms, a 180° rotation around the vertical axis, a 0.5° rotation step, 80 kV, and 125 μA.

### CBCT acquisition

**Figure 1:**
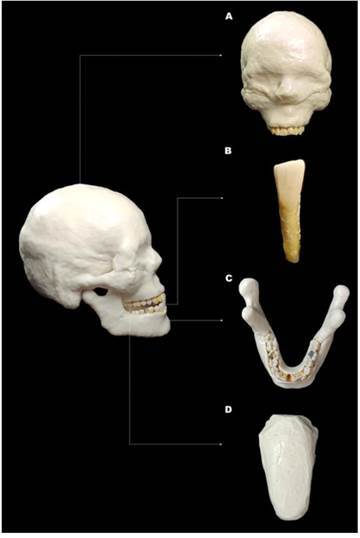
Anthropomorphic phantom consisting of a dry human skull with the mandible, both covered in Mix-D, along with a tongue model made with Mix-D. (A) Frontal view of the dry human skull; (B) Frontal view of the mandibular left central incisor; (C) Occlusal view of the mandible; (D) Tongue model made with Mix-D.

An anthropomorphic model, consisting of a dry human skull and a fully dentate mandible, was used to acquire the CBCT scans. The entire phantom was covered with Mix-D, a soft-tissue simulator material designed to replicate the X-ray attenuation of human soft tissue, as previously described ^25^. Additionally, a tongue model made from the same material was attached to the lingual region of the mandible (Figure 1). Subsequently, the mandibular left central incisor of the dry mandible was carefully removed, allowing the teeth with previously selected long-oval root canals to be individually placed into the empty socket. Each tooth specimen was scanned within the complete phantom using two CBCT devices: VeraView X800 (J Morita, Tokyo, Japan) and OP300 Maxio (Instrumentarium Dental, Tuusula, Finland). The acquisition protocols for each device are detailed in Table 1. Subsequently, the CBCT scans were exported in DICOM format.

**Table 1:** Acquisition parameters of the cone-beam computed tomography dataset

CBCT device	kVp	mA	FOV (cm)	Voxel size (mm^3^)
VeraView X800	70	5	40 x 40	0.08
OP300 Maxio	90	8	50 x 50	0.08

CBCT: cone-beam computed tomography; kVp: kilovoltage-peak; mA: milliampere; FOV: field of view; cm: centimeters; mm^3^: cubic millimeters.

### Micro-CT image evaluation

Each tooth’s images were individually reconstructed using NRecon software (v.1.6.3, Bruker). The micro-CT images, serving as the reference standard, were then analyzed by an experienced operator (F.F.E.T.) possessing extensive experience in micro-CT image evaluation. Each root canal was individually assessed for the detection of voids in the cervical/middle thirds (6 mm) and the apical third (3 mm), using the CTAn software. A score of 0 was assigned if no voids were detected, while a score of 1 was given if voids were present. The same analysis was repeated to assess intra-rater agreement after 30 days.

### CBCT image evaluation

Each CBCT scan was randomly allocated to one of two evaluation phases-either with or without the BAR filter-using the "RAND" function in Excel (Microsoft Co., Redmond, WA, USA). This method ensured that after each evaluation, the CBCT scans received a random code different from their original designation. Evaluations were conducted independently by an experienced oral radiologist (A.F.L.), with over 20 years of practice, and two endodontic specialists (A.O.S.J. and J.C.P.), all highly skilled in image analysis.

Before image analysis, the examiners underwent a calibration process using CBCT scans that displayed voids and void-free regions in various root canal thirds (cervical/middle and apical). These calibration images were not included in the actual assessment. The examiners were blinded to the factors under study, including the type of endodontic sealer and the specific CBCT device used.

Examiners were instructed to realign the multiplanar reconstructions of the CBCT scans along the root’s long axis and to conduct a dynamic examination of each CBCT scan in the axial, sagittal, and coronal planes. All CBCT scans were analyzed using the e-Vol DX software on a 25-inch color LED monitor with a resolution of 2560 x 1080 pixels, in a low-light, quiet room. Examiners were advised to thoroughly evaluate all CBCT scans across the root thirds (i.e., cervical/middle and apical).

To ensure a standardized assessment of root canal fillings, each root was divided into two segments based on the location of the filling material. The first axial reconstruction displaying the filling material in the cervical third and the last axial reconstruction showing it in the apical third were used to determine the root length. This approach allowed for consistent measurement of each root: 6 mm for the cervical/middle thirds and 3 mm for the apical third. By following this method, all observers assessed the root canals in a standardized manner, as described by Fontenele et al. ^26^. Void detection was carried out in two steps:

- Step 1: Examiners could manually adjust the brightness and contrast settings according to their preferences, with the BAR filter deactivated.
- Step 2: Initially, all CBCT scans were systematically evaluated using the five BAR filter intensities (BAR 1 to BAR 5) to thoroughly assess the presence or absence of voids within the filling material. Because different BAR levels can influence subjective visual perception of image quality, one BAR filter intensity was then selected for each CBCT device based on independent assessments and consensus among three calibrated examiners. This selection aimed to obtain the most suitable image in terms of brightness, contrast, and noise, while reducing subjective bias associated with variations in BAR intensity. The BAR 1 filter was selected for the CBCT scans acquired with the OP300 Maxio, whereas the BAR 3 filter was selected for those obtained with the VeraView X800. These selected BAR filter intensities were then consistently applied to all CBCT scans used in the diagnostic performance analysis for detecting voids within filled root canals, ensuring methodological standardization and minimizing multiplicity bias.

Each CBCT scan was evaluated using a 5-point confidence scale to classify voids within the filling material across different root thirds: ^1^ no voids, ^2^ probable no voids, ^3^ uncertain, ^4^ probable presence of voids, and ^5^ presence of voids. To minimize visual fatigue, a maximum of 10 CBCT volumes were evaluated per day, with at least 24 hours between evaluation sessions. To verify intra- and inter-observer reproducibility, 30% of the sample was randomly selected and re-evaluated 30 days after the initial analysis.

**Figure 2:**
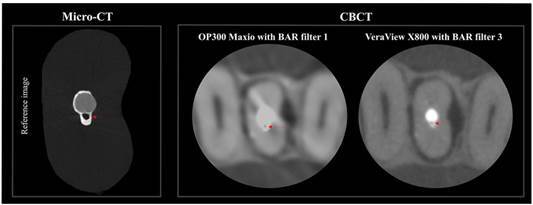
Axial reconstructions of the same specimen obtained using micro-CT as a reference and CBCT scans from the OP300 Maxio (BAR filter intensity 1) and VeraView X800 (BAR filter intensity 3), with red arrows indicating voids within the filling material.

Figure 2 presents an example of a void present within the filling material, comparing the reference image (micro-CT axial reconstruction) with the corresponding visualization on each CBCT device tested (OP300 Maxio and VeraView X800).

### Statistical analysis

Statistical analyses were performed using MedCalc (version 22.0, MedCalc Software Ltd., Ostend, Belgium) and SPSS software (version 24.0, IBM Corp., Armonk, NY). Intra- and inter-observer agreements were evaluated using the weighted Kappa index, interpreted according to the Landis and Koch scale ^27^. Values between 0.00-0.20 indicate slight agreement; 0.21-0.40, fair; 0.41-0.60, moderate; 0.61-0.80, substantial; and 0.81-1.00, almost perfect ^27^. Diagnostic metrics, including the area under the receiver operating characteristic (ROC) curve, sensitivity, and specificity for void detection, were calculated for each factor studied (BAR filter use, type of endodontic sealer, and root canal third) within each CBCT device. Statistical comparisons were made using multi-way analysis of variance (ANOVA) with Tukey's post-hoc test, with a significance level set at 5% for all analyses. A post-hoc power analysis was performed, considering the observed differences and variability among the groups. This analysis produced a statistical power of 92%, indicating a high probability of detecting significant effects within the study’s sample size.

## Results

Regarding intra- and inter-observer reliability, the intra-examiner agreement for evaluating CBCT scans was classified as moderate, with weighted Kappa values ranging from 0.50 to 0.52. The inter-examiner agreement for these scans ranged from moderate (0.47) to almost perfect (0.94). Moreover, the intra-examiner agreement for the micro-CT images, which served as the gold standard, indicated perfect calibration by the examiner, with a weighted Kappa value of 1.00, according to the Landis and Koch scale ^27^. 

The micro-CT assessment of void distribution across different root canal thirds showed a consistent pattern regardless of the sealer used (Table 2). Voids were observed in 70% of the teeth in the cervical/middle thirds and in 60% of the teeth in the apical thirds for both AH Plus Jet and Bio-C Sealer.

**Table 2:** Distribution of teeth (percentage) regarding the presence or absence of voids in different root canal thirds based on sealer type in micro-CT assessment.

Sealer	Cervical/ Middle		Apical	
	Absence	Presence	Absence	Presence
AH Plus Jet (10 Teeth)	3 (30)	7 (70)	4 (40)	6 (60)
Bio-C Sealer (10 Teeth)	3 (30)	7 (70)	4 (40)	6 (60)

**Figure 3:**
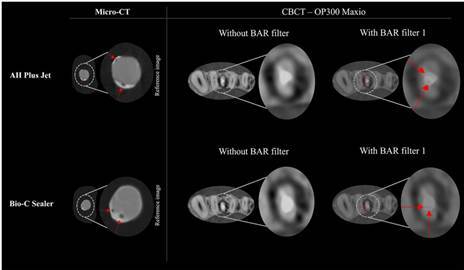
Examples of voids located in the apical third of long-oval root canals filled with AH Plus Jet and Bio-C Sealer. Axial reconstructions of CBCT scans without the BAR filter do not reveal these voids. However, when the BAR filter is applied at intensity level 1, the voids become clearly visible, as indicated by the red arrows. Their presence is confirmed in the reference micro-CT images, where the same regions are also highlighted with red arrows.

Table 3 presents the diagnostic performance metrics, mean area under the ROC curve (AUC), sensitivity, and specificity values, for void detection, categorized by the use of the BAR filter (intensity level 1), sealer type, and root canal third for the OP300 Maxio CBCT device. The use of the BAR filter 1 significantly improved the AUC for detecting voids in the apical root third for both AH Plus Jet and Bio-C Sealer (p<0.05). Without the BAR filter, both AH Plus Jet and Bio-C Sealer had an AUC value of 0.53 in the apical third. When the BAR filter 1 was applied, the AUC values significantly increased, with AH Plus Jet reaching 0.72 and Bio-C Sealer reaching 0.65 (p<0.05) (Figure 3). This improvement was statistically significant depending on the root third assessed for both sealers (p<0.05). Specifically, the apical third (AUC of 0.72 for AH Plus Jet and 0.65 for Bio-C Sealer) showed higher AUC values compared to the cervical/middle root thirds (AUC of 0.61 for AH Plus Jet and 0.53 for Bio-C Sealer). Sensitivity values were not significantly influenced by the factors investigated (p>0.05). However, specificity values were influenced by the type of sealer when using the BAR filter 1 in the apical third (p<0.05). In this context, AH Plus Jet demonstrated higher specificity (1.00) compared to Bio-C Sealer (0.42).

**Table 3:** Mean ± standard deviation (SD) of the area under the receiver operating curve (AUC), sensitivity, and specificity values for the detection of voids based on BAR use (BAR filter intensity level 1), sealer, and root third in the OP300 Maxio CBCT device.

BAR	Sealer	Root third	AUC	Sensitivity	Specificity
Without BAR	AH Plus Jet	Cervical/Middle	0.60 (0.01)	0.39 (0.00)	0.86 (0.01)
		Apical	0.53 (0.02)	0.40 (0.34)	0.80 (0.35)
	Bio C-Sealer	Cervical/Middle	0.57 (0.06)	0.73 (0.14)	0.50 (0.00)
		Apical	0.53 (0.02)	0.45 (0.48)	0.58 (0.29)
With BAR filter 1	AH Plus Jet	Cervical/Middle	0.61 (0.03)	0.63 (0.21)	0.67 (0.17)
		Apical	0.72 (0.07) *#	0.47 (0.11)	1.00 (0.00) +
	Bio C-Sealer	Cervical/Middle	0.53 (0.00)	0.67 (0.04)	0.50 (0.00)
		Apical	0.65 (0.03) *#	1.00 (0.00)	0.42 (0.14)

# indicates a statistically significant difference in the use of BAR within the sealer and root third investigated (p<0.05).

* indicates a statistically significant difference between the root thirds within both sealer and BAR conditions (p<0.05).

+ indicates a statistically significant difference between the sealers within both root third and BAR conditions (p<0.05).

Table 4 presents the diagnostic performance for void detection according to the factors studied for the VeraView X800 CBCT device. The AUC and sensitivity values were not significantly influenced by the use of the BAR filter (intensity level 3), sealer type, or root third (p>0.05). However, the use of the BAR filter 3 significantly improved the specificity for detecting voids in the cervical/middle root thirds when Bio-C Sealer was present (p<0.05). Specifically, with the BAR filter 3, the specificity value increased from 0.58 to 1.00 (Figure 4).

**Table 4:** Mean ± standard deviation (SD) of the area under the receiver operating curve (AUC), sensitivity, and specificity values for the detection of voids based on BAR use (BAR filter intensity level 3), sealer, and root third in the VeraView X800 CBCT device

BAR	Sealer	Root third	AUC	Sensitivity	Specificity
Without BAR	AH Plus Jet	Cervical/Middle	0.67 (0.08)	0.51 (0.17)	0.80 (0.00)
		Apical	0.62 (0.04)	0.60 (0.20)	0.70 (0.10)
	Bio C-Sealer	Cervical/Middle	0.56 (0.04)	0.61 (0.29)	0.58 (0.29)
		Apical	0.54 (0.04)	0.56 (0.20)	0.50 (0.00)
With BAR filter 3	AH Plus Jet	Cervical/Middle	0.66 (0.10)	0.64 (0.27)	0.67 (0.11)
		Apical	0.56 (0.00)	0.40 (0.00)	0.80 (0.00)
	Bio C-Sealer	Cervical/Middle	0.82 (0.17)	0.66 (0.31)	1.00 (0.00) #
		Apical	0.72 (0.16)	0.61 (0.38)	0.83 (0.14)

# indicates a statistically significant difference in the use of BAR within the sealer and root third investigated (p<0.05).

**Figure 4:**
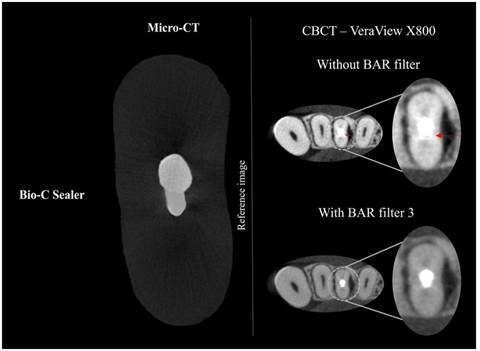
An example of an axial reconstruction of the CBCT scan showing a false-positive detection of a void in the cervical/middle thirds of a canal filled with Bio-C Sealer, as indicated by the red arrow. After applying the BAR filter at intensity level 3, the absence of the void was confirmed, as also seen in the reference micro-CT image.

## Discussion

Detecting voids in root canal fillings using CBCT scans is challenging in clinical practice, primarily due to blooming artifacts, which result from beam-hardening and scattered radiation interacting with high-density objects ^6^ ^,^ ^28^. These artifacts create hyperdense halos around high-density materials such as gutta-percha, endodontic sealers, and intracanal metal posts, leading to volumetric distortions that can either underestimate or overestimate the actual dimensions of the scanned object on CBCT images ^28^. As a result, this distortion may give the false impression that the root canal is fully filled ^6^ ^,^ ^10^ ^,^ ^12^ ^,^ ^28^. This study is the first to evaluate the effect of the BAR filter on void detection in long-oval root canals of mandibular incisors filled with two endodontic sealers of differing radiopacity. The findings were promising, indicating that the BAR filter significantly enhanced diagnostic accuracy for void detection across various conditions, ultimately refuting the null hypothesis.

The current findings indicate that applying the BAR filter 1 to CBCT scans obtained with the OP300 Maxio device significantly improved the accuracy of void detection in the apical third of root canals filled with AH Plus Jet and Bio-C Sealer. With the BAR filter 1 enabled, the AUC values for detecting voids in the apical third of root canals increased from 0.53 to 0.72 for AH Plus Jet and from 0.53 to 0.65 for Bio-C Sealer, approaching the ideal AUC values of 1.0, which signifies perfect accuracy (Figure 3). This improvement can be attributed to the enhanced grayscale contrast provided by the BAR filter ^16^ ^,^ ^17^ ^,^ ^18^ ^,^ ^19^, which significantly reduces blooming artifacts caused by intracanal materials (i.e., high-density materials) such as endodontic sealer and gutta-percha. However, the detection of tiny voids remains inherently limited by the spatial resolution of CBCT scans. In this study, both CBCT devices operated with a voxel size of 0.08 mm³, meaning that voids smaller than this threshold cannot be detected because of the partial volume effect. Previous studies have shown that small voids clearly detected in micro-CT ^6^ or nano-CT ^12^ images often remain undetectable in CBCT scans due to blooming artifacts and lower spatial resolution. For this reason, the current study intentionally retained all voids identified by micro-CT, including those below the CBCT detection limit, to preserve clinical representativeness and avoid artificially inflating CBCT diagnostic performance.

In addition, the current study showed that the specificity values for void detection with the OP300 Maxio device varied depending on the type of endodontic sealer used. AH Plus Jet, with higher radiopacity, achieved a higher specificity (1.00) compared to Bio-C Sealer (0.42) in the apical third of the root canals. This finding is clinically significant because root canals filled with AH Plus Jet are more prone to blooming artifacts due to their high radiopacity, which can make void detection challenging ^6^ ^,^ ^10^ ^,^ ^11^ ^,^ ^12^. The increased specificity for AH Plus Jet indicates that examiners were better able to accurately identify voids in the apical third, thereby reducing the risk of false positives and avoiding unnecessary interventions, such as endodontic retreatment or tooth extraction ^6^. Therefore, using the BAR filter 1 to evaluate CBCT scans of teeth filled with AH Plus Jet, particularly when acquired with the OP300 Maxio device, can significantly enhance diagnostic accuracy. This improvement enables clinicians to more accurately identify voids within the filling material, reducing the risk of misdiagnosis and unnecessary treatments, ultimately leading to better endodontic outcomes.

The diagnostic performance varied across different regions of the root canal when the BAR filter 1 was applied to the OP300 Maxio scans. AUC values were higher in the apical third compared to the cervical/middle thirds, regardless of the endodontic sealer. These differences can be attributed to the distinct cross-sections of the long-oval root canals used in this study. The apical third has a smaller volume than the cervical/middle thirds, resulting in a reduced amount of sealer ^2^ ^,^ ^3^. Consequently, blooming artifacts may have caused a greater overestimation of sealer volume in the cervical/middle thirds ^6^ ^,^ ^10^ ^,^ ^11^ ^,^ ^12^. The increased amount of endodontic sealer in these areas may have limited the diagnostic performance for void detection, even with the BAR filter 1 applied, as the material expansion may have masked voids. 

Nonetheless, these findings are clinically significant because the apical third is the most critical zone of the root canal due to its complex anatomy, which includes ramifications, lateral canals, and the apical delta, complicating adequate preparation, disinfection, and obturation ^29^ ^,^ ^30^. This effect of the root canal region was not observed in images without the BAR filter, likely due to the higher expression of artifacts throughout the root canal, regardless of region, which compromised image quality across the entire root. 

CBCT scans acquired with the VeraView X800 device showed no significant influence from the variables analyzed in this study on AUC and sensitivity values. This may be because these diagnostic metrics were consistently high for the VeraView X800, regardless of the BAR filter, endodontic sealer, or root canal third, likely due to the physical and technical characteristics unique to each CBCT device ^31^. In this study, all CBCT scans were obtained using a high-resolution protocol with a smaller voxel size (0.08 mm³) and the smallest available FOV on both devices (40 x 40 mm for the VeraView X800 and 50 x 50 mm for the OP300 Maxio). Previous studies have demonstrated that blooming artifacts are influenced by several factors, including voxel size, kilovoltage, tube current, detector pixel size, and FOV size ^6^ ^,^ ^10^ ^,^ ^11^. 

The VeraView X800 device had a smaller FOV (40 x 40 mm) and a lower tube current (5 mA) compared to the OP300 device, which had an FOV of 50 x 50 mm and a tube current of 8 mA. A smaller FOV and lower mA are associated with fewer blooming artifacts on CBCT scans of filled teeth ^10^, which may explain why the study factors did not significantly impact the VeraView X800 images. 

In contrast, applying the BAR filter 3 to CBCT scans from the VeraView X800 device significantly increased specificity values (from 0.58 to 1.00) for detecting voids in the cervical/middle thirds of samples filled with Bio-C Sealer. This improvement indicates that the BAR filter enhanced examiners' ability to accurately confirm the absence of voids (Figure 4), which is crucial in clinical scenarios where beam hardening artifacts, such as hypodense streaks, could otherwise lead to false positives. These findings are clinically important because accurate void detection on CBCT scans is essential for diagnosing potential treatment failures and planning effective endodontic retreatments, especially given the frequent use of bioceramic sealers in endodontic practice.

In this study, micro-CT images served as a reference standard for detecting voids and validating CBCT scans analysis results. This choice was strategically based on previous research demonstrating the precision of micro-CT in assessing endodontic filling quality ^1^ ^,^ ^2^ ^,^ ^3^ ^,^ ^6^ ^,^ ^10^ ^,^ ^11^ ^,^ ^12^. Different sealers can produce varying levels of blooming artifacts on CBCT scans, which may significantly compromise or even prevent accurate void detection in the filling material ^11^. However, it is important to note that micro-CT is limited to laboratory settings and is not applicable in clinical contexts ^12^. This study is notable for its pioneering approach, demonstrating the effectiveness of a post-processing filter applied to CBCT scans to improve void detection under specific imaging conditions. The micro-CT analysis of void distribution across different root canal thirds revealed a consistent pattern regardless of the sealer used, with a high incidence of voids in both the cervical/middle thirds (70%) and the apical third (60%), aligning with previous findings ^2^ ^,^ ^3^. 

To achieve research objectives, an anthropomorphic phantom was constructed for ex vivo applications, consisting of a dry human skull and mandible covered with Mix-D material, along with a tongue model also made of Mix-D ^25^. This setup was designed to simulate the X-ray attenuation of facial soft tissues during CBCT scans acquisition, closely replicating a realistic clinical scenario ^25^ ^,^ ^32^ ^,^ ^33^. Additionally, mandibular central incisors with a long-oval cross-section were selected in this study due to their challenging morphology for obturation. The extensive buccolingual dimension of these canals can compromise the three-dimensional filling of the root canal system, negatively affecting the prognosis of endodontic treatment ^2^ ^,^ ^3^ ^,^ ^4^. The selection of this anatomy aims to ensure the clinical relevance of the findings, aligning them with real clinical scenarios where root canals exhibit a non-circular cross-section, whose complexity directly impacts filling effectiveness ^2^ ^,^ ^3^. However, certain limitations of the study should be noted. Firstly, patient movement was not considered, which can result in motion artifacts commonly observed in CBCT scans, potentially reducing image quality and diagnostic accuracy ^30^. Secondly, the images were acquired from only two CBCT devices, which may limit the generalizability of the results to other units. Thus, the current findings should be interpreted with caution. Further studies are recommended to investigate the impact of the BAR filter on void detection in root canals with varying anatomical complexities. Additionally, it is important to test the BAR filter in canals filled using different obturation techniques, such as lateral compaction and the Tagger hybrid technique, as well as with other commercially available sealers. 

The current findings are of significant clinical importance, indicating that the BAR filter enhances diagnostic accuracy in visualizing voids within root canal filling on CBCT scans. This advancement addresses recurring challenges noted in the literature related to this diagnostic task ^6^ ^,^ ^12^. The findings are pivotal for enabling a safer and more reliable decision-making process in endodontic practice, allowing clinicians to assess root canal obturation quality and investigate treatment failures more precisely. This approach may lead to more effective retreatments and reduce iatrogenic errors, potentially decreasing unnecessary tooth extractions. 

It is important to emphasize that recommending a CBCT scan is justified only when periapical radiographs yield inconclusive or clinically incompatible findings, in line with guidelines from the American Association of Endodontists (AAE) and the European Commission on Radiological Protection ^34^ ^,^ ^35^. The high diagnostic complexity in cases of suspected endodontic treatment failures is widely discussed in the literature, underscoring the need to assess the quality of root canal obturation to detect potential voids. In such specific clinical scenarios, the use of CBCT scans is essential for accurate analysis ^34^ ^,^ ^35^.

## Conclusion

In conclusion, the diagnostic performance of the BAR filter varies depending on the specific CBCT device and the endodontic sealer used. The BAR filter at intensity level 1 significantly improved diagnostic performance for void detection in the apical third of long-oval root canals when used with the OP300 CBCT unit and both AH Plus Jet and Bio-C Sealer. In contrast, while the VeraView X800 device did not show an overall improvement in diagnostic performance with the BAR filter, the BAR filter at intensity level 3 achieved a significant increase in specificity for detecting voids in the cervical/middle thirds of root canals filled with Bio-C Sealer.

## Data Availability

The data that support the findings of this study are available from the corresponding author upon reasonable request.
